# The Prevalence of Vitamin B12 Deficiency Among Diabetic Patients Who Use Metformin

**DOI:** 10.7759/cureus.74559

**Published:** 2024-11-27

**Authors:** Ahmad Aboshaiqah, Bader Aboshaiqah, Naif M Alharbi, Tariq I Almunyif, Saad D Binghanim, Abdullah K Almejalli

**Affiliations:** 1 College of Nursing, King Saud University, Riyadh, SAU; 2 Department of Medicine, King Saud Bin Abdulaziz University for Health Sciences College of Medicine, Riyadh, SAU; 3 Department of Medicine, King Saud Bin Abdulaziz University for Health Sciences College of Medicine, Riyadh , SAU

**Keywords:** diabetes, metformin, prevalence, type 2 diabetes, vitamin b12 deficiency

## Abstract

Background and aim: Vitamin B12 deficiency is a recognized concern among patients with type 2 diabetes mellitus (T2DM) using metformin due to its potential impact on health outcomes. This study investigates the prevalence of vitamin B12 deficiency among diabetic patients using metformin at Dr. Sulaiman Al Habib Medical Group (HMG) hospitals.

Methods: This retrospective cohort study utilized electronic medical records from the VIDA system (www.cloudsolutions.com) over a one-year period. Participants included adult T2DM patients managed with metformin therapy. Data collection involved demographic details, medical history, medication usage, and serum vitamin B12 levels. Statistical analyses included descriptive statistics and comparative tests to assess deficiency prevalence across demographics.

Results: Among the study population (N=37,781), vitamin B12 deficiency prevalence ranged from 460 (4.6%) to 507 (5.5%) across four quarters of 2023. Females exhibited a higher prevalence than males (P < 0.05), particularly in the second and fourth quarters. Age-related analyses identified younger and middle-aged groups (≤25 years and 36-45 years) as having higher deficiency rates (P < 0.001). Medication use, specifically metformin, correlated with increased deficiency prevalence (P < 0.001).

Discussion: The findings underscore the significance of gender, age, and medication use in influencing vitamin B12 status among diabetic patients using metformin. The study highlights the need for tailored monitoring strategies and interventions to mitigate deficiency risks and optimize patient outcomes. Future research should explore mechanisms underlying these associations and expand findings to broader populations.

Conclusion: This study provides critical insights into the epidemiology of vitamin B12 deficiency among diabetic patients using metformin at HMG hospitals. The results emphasize the importance of personalized healthcare approaches to address nutrient deficiencies and improve overall patient care. This study summarizes the key elements of your research paper, focusing on the prevalence of vitamin B12 deficiency in relation to metformin use among diabetic patients.

## Introduction

The word "cobalamin" refers broadly to substances with physiologic vitamin B12 action. These substances have a role in myelin production and repair, methyl transfer, and nucleic acid metabolism [1]. They are essential for appropriate red blood cell production and healthy brain function. Meats (particularly beef, hog, and organ meats (such as liver)), poultry, eggs, fortified cereals, milk and milk products, and shellfish, including clams, oysters, mackerel, and salmon, are all good sources of vitamin B12. Food-bound vitamin B12, which is bound to the R protein (haptocorrin), is released in the acidic environment of the stomach. In the small intestine, pancreatic enzymes break down the B12 complex (B12-R protein). Intrinsic factor, which is released by parietal cells in the gastrointestinal mucosa, cleaves and binds to vitamin B12 thereafter. Vitamin B12 is absorbed in the terminal ileum, where an intrinsic factor is necessary [2].

Common symptoms encompass easy fatigue upon exertion, palpitations, and a pale skin complexion. Reports have also noted occurrences of skin hyperpigmentation, glossitis, and infertility. The progression of demyelination is responsible for neurological manifestations, which may involve peripheral neuropathy, loss of reflexes (areflexia), and a decline in proprioception and vibratory sensation. In spite of the importance of vitamin B12, there is still insufficiency due to poor consumption and malabsorption. Individuals following strict vegetarian or vegan diets may have a lower intake of vitamin B12 since it's mainly found in animal-based foods [3]. The clinical presentations exhibit a wide array of forms and degrees, encompassing less severe symptoms like fatigue, sensory neuropathy, atrophic glossitis (referred to as Hunter's glossitis), and isolated occurrences of macrocytosis or exaggerated neutrophil segmentation. On the more severe end, the manifestations can include conditions like spinal cord combined sclerosis, hemolytic anemia, and in extreme cases, pancytopenia [4]. Due to the significant storage of vitamin B12 in the liver, the emergence of clinical symptoms can be postponed by as much as a decade following the beginning of deficiency [5,6]. If neuronal loss happens in the posterior and lateral spinal cord tracts, areflexia can become permanent. In instances of more severe and prolonged deficiency, a dementia-like condition, accompanied by episodes of psychosis, is feasible [5-7].

The absorption of vitamin B12 relies on various processes, one of which is the production of intrinsic factor (IF). When the production of gastric intrinsic factor is compromised, as seen in situations like the destruction of gastric parietal cells during gastritis or a significant reduction of these cells, as in the case of gastric bypass surgery, the result is a decreased ability to absorb vitamin B12. Vitamin B12 uptake primarily occurs in the distal ileum. If there's an issue like ileal dissection, bacterial overgrowth, or intestinal disorders such as Crohn's disease, this can lead to a decreased capacity of the ileal cells to absorb vitamin B12, consequently reducing its intake. Additionally, medications that control the secretion of gastric acid production, like proton pump inhibitors, can also contribute to a deficiency in vitamin B12 due to the impaired release of vitamin B12 from dietary proteins [2].

The increasing prevalence of type 2 diabetes is a significant concern in the global healthcare landscape. Data on global and regional trends in type 2 diabetes across all age groups from 1990 to 2017 were collected, and predictive estimates were generated using the SPSS Time Series Modeler. In 2017, roughly 462 million people were affected by type 2 diabetes, constituting approximately 6.28% of the world's population. This prevalence was further broken down into 4.4% for individuals aged 15 to 49 years, 15% for those aged 50 to 69, and 22% for those aged 70 and older, resulting in a prevalence rate of 6059 cases per 100,000 individuals. Alarmingly, more than one million deaths annually can be attributed to diabetes alone, ranking it as the ninth leading cause of death [8]. The global burden of diabetes mellitus is on the rise, with developed regions like Western Europe experiencing a particularly rapid increase [9]. The incidence of diabetes is equally distributed between genders, with the highest occurrence observed at approximately 55 years of age. Projections indicate that the global prevalence of type 2 diabetes is expected to climb to 7079 cases per 100,000 individuals by 2030, indicating a continued upward trend across all regions of the world [10].

Several studies have investigated the prevalence of vitamin B12 deficiency among individuals with type 2 diabetes who use metformin. In a cross-sectional study by Al Saeed and Baraja [11] involving 307 metformin-treated diabetic patients in Saudi Arabia, a 9.4% prevalence of vitamin B12 deficiency was reported. Factors contributing to this deficiency included patient age, female gender, low socioeconomic status, and prolonged use of metformin. The study also highlighted associated health issues such as anemia, fatigue, weakness, and nerve damage due to vitamin B12 deficiency, stressing the importance of early detection and preventive measures. Kakarlapudi et al. [12] conducted a systematic review of 31 studies with 12,314 participants, confirming a significant decrease in serum vitamin B12 levels associated with metformin use, especially with prolonged and higher doses. Similarly, Khattab et al. [13] reviewed 19 relevant studies and concluded that metformin use heightens the risk of vitamin B12 deficiency, which correlates with elevated homocysteine and reduced folate levels. They recommended regular monitoring of vitamin B12 levels during metformin treatment to mitigate these risks. These findings emphasize the necessity for diligent monitoring and management strategies in patients with type 2 diabetes using metformin to prevent potential complications stemming from vitamin B12 deficiency.

The mechanisms underlying vitamin B12 deficiency caused by long-term use of metformin are not yet well understood. However, the primary mechanisms involve alterations in the absorption and metabolism of vitamin B12. These mechanisms include metformin traveling to the liver and causing increased accumulation of vitamin B12, leading to decreased distribution in tissues and altered metabolism of the vitamin; interference with the binding of the IF-vitamin B12 complex to cubilin receptors on the intestinal villi and enterocytes in the ileum; reduced secretion of intrinsic factor (IF) by stomach parietal cells; impairment of the enterohepatic recycling of vitamin B12 due to alterations in the metabolism and reabsorption of bile acids; and reduced motility of the small intestine, which results in bacterial overgrowth and prevents the IF-vitamin B12 complex from being absorbed in the distal ileum [14]. The prevalence of vitamin B12 deficiency was (3.6%), and the majority of the sample had borderline B12 levels (66.1%). The vitamin B12 deficiency and borderline levels were strongly associated with the dose of metformin. Patients taking doses of metformin more than 1000 mg had lower levels of vitamin B12. The use of multivitamins and vitamin B complex was assessed, and it was found that there was a marked decrease in the prevalence of vitamin B12 deficiency in patients using vitamin B complex containing more than 200 mcg of vitamin B12 [15].

Metformin continues to be the primary choice among oral medications for managing type 2 diabetes due to its established track record of long-term safety and effectiveness. In fact, it holds the distinction of being the most commonly prescribed oral insulin-sensitizing medication, with over 100 million individuals worldwide receiving prescriptions. This includes individuals with conditions like prediabetes, insulin resistance, and polycystic ovary syndrome. However, there is evidence indicating that prolonged and high-dose metformin usage can have adverse effects on vitamin B12 levels. Vitamin B12, also known as cobalamin, is a water-soluble vitamin primarily sourced from animal-based foods. At the cellular level, vitamin B12 serves as a cofactor for enzymes crucial in DNA synthesis and neuroprotection. Nonetheless, there are currently no definitive guidelines available for screening for vitamin B12 deficiency in individuals undergoing metformin therapy, and this deficiency often goes undetected in such patients [14,15].

The studies have significantly contributed to understanding the relationship between metformin medication and vitamin B12, yet they reveal several gaps that warrant further investigation. Many studies have primarily focused on the short-term effects of metformin as a risk factor for vitamin B12 deficiency. This highlights the need for additional research to comprehensively explore the long-term impacts of metformin on B12 levels and to reveal the underlying mechanisms involved. Research should also investigate dose-response relationships to determine thresholds at which the risk of deficiency increases significantly, facilitating personalized treatment approaches. Understanding how different age groups, ethnicities, and geographical regions may vary in vulnerability to B12 deficiency is crucial for developing tailored interventions. Furthermore, conducting trials to assess intervention effectiveness, qualitative research to capture patient perspectives, and comprehensive studies on the broad impact of long-term B12 deficiency in metformin users are essential for advancing our knowledge and improving clinical outcomes.

## Materials and methods

Study settings and participants

The research was conducted at Dr. Sulaiman Al Habib Medical Group (HMG) hospitals using the VIDA system (www.cloudsolutions.com), an electronic medical records (EMR) platform, over a span of one year. The primary objective of this retrospective cohort study was to investigate the prevalence of vitamin B12 deficiency among patients diagnosed with type 2 diabetes mellitus (T2DM) who received metformin therapy and within the hospital network. Inclusion and exclusion criteria for study subjects are demonstrated in Table 1.

**Table 1:** Inclusion and exclusion criteria for study subjects

Criteria Type	Criteria Description
Inclusion	Adult patients (>18 years old)
Inclusion	Both male and female
Inclusion	Patients with type 2 diabetes who are being managed with metformin
Exclusion	Any patients with other medical conditions affects B12 absorption
Exclusion	Patients who are using vitamin B12 supplements or multivitamin tablets

Dr. Sulaiman Al Habib Medical Group hospitals were selected as the study setting due to their extensive healthcare services and accessibility to comprehensive patient records. The study population comprised adult patients aged 18 years and above with a confirmed diagnosis of type 2 diabetes mellitus. Eligible participants were identified from electronic medical records (EMRs) of individuals actively managed with metformin therapy for T2DM during the study period.

Data collection method

Data collection involved a thorough review of EMRs using the VIDA system. A total of 37,781 patients were selected and studied. A structured data collection form was designed to systematically extract pertinent information, including patient demographics (age, gender), medical history (diabetes duration, comorbidities), medication usage (metformin, vitamin B12 supplementation), and laboratory results (serum vitamin B12 levels). The use of predefined criteria ensured consistency and accuracy in data extraction from electronic records, eliminating potential biases associated with subjective methods. 

Statistical analysis

The statistical analysis aimed to evaluate the prevalence of vitamin B12 deficiency among distinct patient subgroups, including those managed with metformin therapy for type 2 diabetes mellitus within HMG hospitals. Descriptive statistics such as means, frequencies, and percentages were employed to summarize patient demographics, clinical characteristics, and vitamin B12 deficiency rates. Comparative analyses utilized appropriate statistical tests (e.g., Chi-square tests, t-tests) to assess differences in vitamin B12 deficiency prevalence between patient subgroups, establishing associations between metformin use, and vitamin B12 status.

Ethical considerations were paramount throughout the study, with necessary approvals obtained from the Institutional Review Board (IRB) of Dr. Sulaiman Al Habib Medical Group to ensure patient confidentiality and data security. All data analyses were conducted using anonymized patient information to safeguard privacy and adhere to institutional regulations.

In summary, this retrospective cohort study leveraged the VIDA system to investigate vitamin B12 deficiency prevalence in patients with type 2 diabetes mellitus managed with metformin therapy within Dr. Sulaiman Al Habib Medical Group hospitals. The robust data collection method and rigorous statistical analysis provided valuable insights into potential associations between metformin use and vitamin B12 deficiency, contributing to evidence-based healthcare practices and patient management strategies within the hospital network.

## Results

Table 2 presents sociodemographic characteristics, including gender, age, and vitamin B12 status, across four quarters of the year 2023. Gender distribution shows a slightly higher percentage of females than males across all quarters except quarter 4, with females ranging between 4616 (49.5%) to 4756 (51.8%) and males ranging between 4423 (48.2%) to 4700 (50.5%). Age distribution reveals the largest age group to be 56-65 years, ranging between 3175 (33.9%) in quarter 2 to 3379 (36.3%) in quarter 4. The ≤25 years age group is the smallest, accounting for not more than 123 (1.3%) in any quarter. The majority of the patients were Saudi citizens, ranging between 8204 (89.4%) to 9021 (91%). Vitamin B12 status indicates that the majority of the participants have normal levels, ranging between 8672 (94.5%) to 9453 (95.4%), while a smaller proportion is deficient.

**Table 2:** Sociodemographic characteristics per quarter of the year 2023

		Q1 2023		Q2 2023		Q3 2023		Q4 2023	
		N	%	N	%	N	%	N	%
Gender	Female	4756	51.8	4759	50.8	4967	50.1	4616	49.5
	Male	4423	48.2	4614	49.2	4946	49.9	4700	50.5
	Total	9179	100.0	9373	100.0	9913	100.0	9316	100.0
Age	≤25 years	123	1.3	102	1.1	118	1.2	86	.9
	26-35 years	554	6.0	579	6.2	541	5.5	448	4.8
	36-45 years	1238	13.5	1363	14.5	1295	13.1	1083	11.6
	46-55 years	1752	19.1	1765	18.8	1842	18.6	1768	19.0
	56-65 years	3138	34.2	3175	33.9	3503	35.3	3379	36.3
	>65 years	2374	25.9	2389	25.5	2614	26.4	2552	27.4
	Total	9179	100.0	9373	100.0	9913	100.0	9316	100.0
Nationality	Saudi	8204	89.4	8397	89.6	9021	91.0	8418	90.4
	Non-Saudi	975	10.6	976	10.4	892	9.0	898	9.6
	Total	9179	100.0	9373	100.0	9913	100.0	9316	100.0
Vitamin B12	Normal	8672	94.5	8900	95.0	9453	95.4	8830	94.8
	Deficiency	507	5.5	473	5.0	460	4.6	486	5.2
	Total	9179	100.0	9373	100.0	9913	100.0	9316	100.0
Using Medication	No	2599	28.3	2953	31.5	3247	32.8	3052	32.8
	Yes	6580	71.7	6420	68.5	6666	67.2	6264	67.2
	Total	9179	100.0	9373	100.0	9913	100.0	9316	100.0

**Figure 1:**
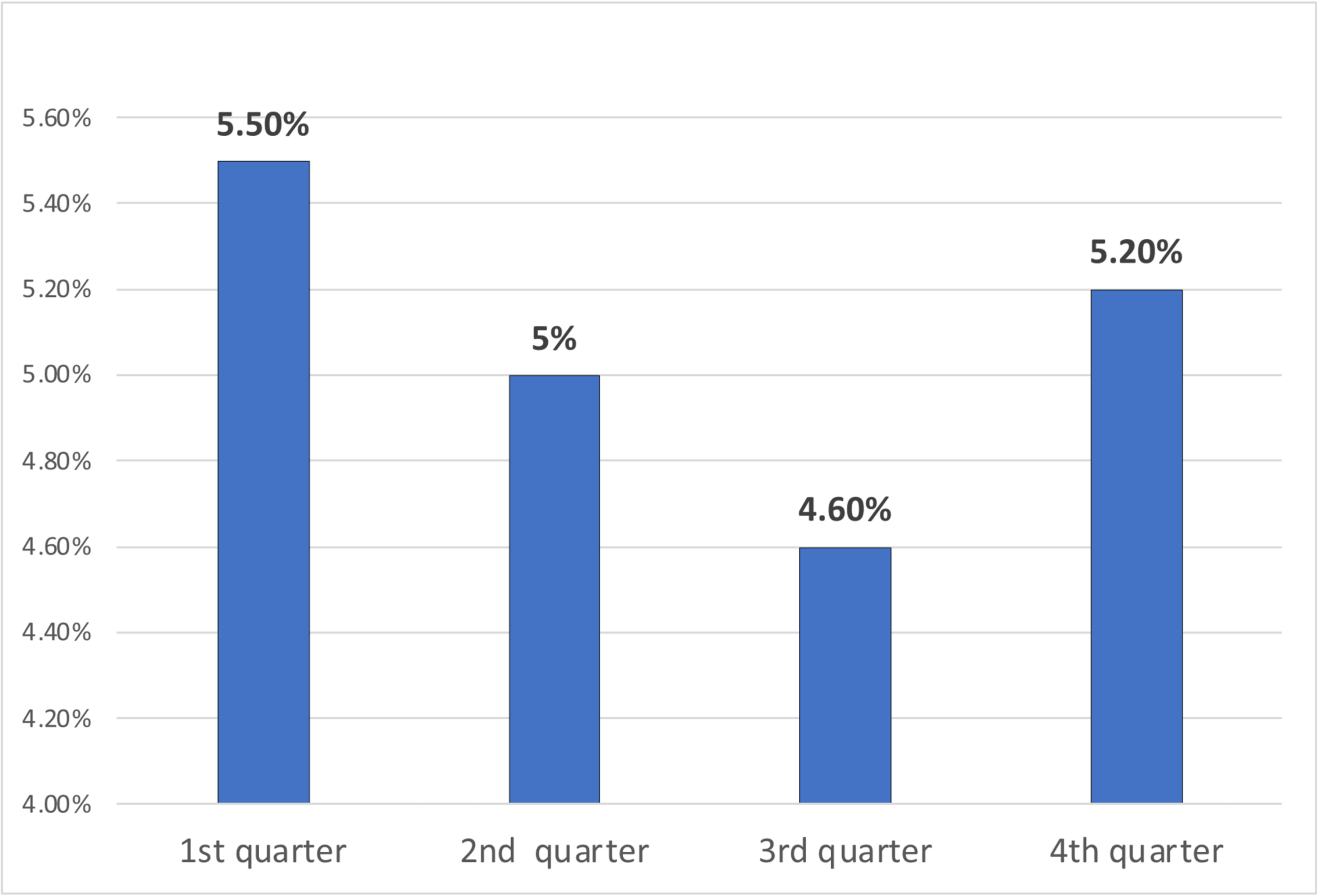
The prevalence of vitamin B12 deficiency per quarter of the year 2023

The prevalence of vitamin B12 deficiency was 5.5%,, 5%, 4.6%, and 5.2% in the 1st, 2nd, 3rd and 4th quarters respectively (Figure 1).

Table 3 illustrates the relationship between vitamin B12 levels and gender across four quarters. Females consistently exhibited a higher proportion in the deficiency category in all four quarters. However, the difference in female and male proportion is not significant in quarters 1 and 3, whereas in the first quarter, the findings were reported as 277 (5.8%) and 230 (5.2%) in females and males, respectively (p=0.191) and similarly 235 (4.7%) vs 225 (4.5%) for females and males, respectively (p=0.667). However, the proportion of females was reported to be significantly higher than males in quarter 2 and quarter 4. In quarter 2, females were 273 (5.7%) vs 200 (4.3%) males with p value=0.002, and similar kind of findings were reported as 269 (5.8%) and 217 (4.6%) in females and males respectively (p=0.009).

**Table 3:** Relationship between vitamin B12 and study participant's gender in all quarters of the year 2023

Quarters	Vitamin B12		Gender		Total	P value
			Female	Male		
1^st^ quarter	Normal	N	4479	4193	8672	0.191
		%	94.2%	94.8%	94.5%	
	Deficiency	N	277	230	507	
		%	5.8%	5.2%	5.5%	
2^nd^ quarter	Normal	N	4486	4414	8900	0.002
		%	94.3%	95.7%	95.0%	
	Deficiency	N	273	200	473	
		%	5.7%	4.3%	5.0%	
3^rd^ quarter	Normal	N	4732	4721	9453	0.667
		%	95.3%	95.5%	95.4%	
	Deficiency	N	235	225	460	
		%	4.7%	4.5%	4.6%	
4^th^ quarter	Normal	N	4347	4483	8830	0.009
		%	94.2%	95.4%	94.8%	
	Deficiency	N	269	217	486	
		%	5.8%	4.6%	5.2%	

Table 4 displays the relationship between vitamin B12 levels and age groups across four quarters. It was observed that the age groups of <=25 years and 36-45 years consistently exhibited significantly higher prevalence rates of vitamin B12 deficiency compared to other age groups (0<0.001).

**Table 4:** Relationship between vitamin B12 and participant's age in all quarters of the year 2023

Quarters	Vitamin B12 Status	Age in years							P value
		≤25	26-35	36-45	46-55	56-65	>65	total	
1^st^ quarter	Normal	106	497	1135	1657	3018	2259	8672	<0.001
		86.2%	89.7%	91.7%	94.6%	96.2%	95.2%	94.5%	
	Deficiency	17	57	103	95	120	115	507	
		13.8%	10.3%	8.3%	5.4%	3.8%	4.8%	5.5%	
2^nd^ quarter	Normal	93	519	1286	1672	3061	2269	8900	<0.001
		91.2%	89.6%	94.4%	94.7%	96.4%	95.0%	95.0%	
	Deficiency	9	60	77	93	114	120	473	
		8.8%	10.4%	5.6%	5.3%	3.6%	5.0%	5.0%	
3^rd^ quarter	Normal	112	484	1209	1770	3373	2505	9453	<0.001
		94.9%	89.5%	93.4%	96.1%	96.3%	95.8%	95.4%	
	Deficiency	6	57	86	72	130	109	460	
		5.1%	10.5%	6.6%	3.9%	3.7%	4.2%	4.6%	
4^th^ quarter	Normal	83	414	1000	1675	3210	2448	8830	<0.001
		96.5%	92.4%	92.3%	94.7%	95.0%	95.9%	94.8%	
	Deficiency	3	34	83	93	169	104	486	
		3.5%	7.6%	7.7%	5.3%	5.0%	4.1%	5.2%	

The comparison of vitamin B12 deficiency between Saudi and non-Saudi nationalities is given in Table 5. There was no significant difference in the prevalence of normal vitamin B12 levels between Saudi and non-Saudi nationals across all quarters (p > 0.05).

**Table 5:** Relationship between vitamin B12 and participant's nationality in all quarters of the year 2023

Quarters	Vitamin B12		Nationality		Total	P value
			Saudi	Non-Saudi		
1^st^ quarter	Normal	N	7746	926	8672	0.472
		%	94.4%	95.0%	94.5%	
	Deficiency	N	458	49	507	
		%	5.6%	5.0%	5.5%	
2^nd^ quarter	Normal	N	7969	931	8900	0.511
		%	94.9%	95.4%	95.0%	
	Deficiency	N	428	45	473	
		%	5.1%	4.6%	5.0%	
3^rd^ quarter	Normal	N	8602	851	9453	0.948
		%	95.4%	95.4%	95.4%	
	Deficiency	N	419	41	460	
		%	4.6%	4.6%	4.6%	
4^th^ quarter	Normal	N	7973	857	8830	0.356
		%	94.7%	95.4%	94.8%	
	Deficiency	N	445	41	486	
		%	5.3%	4.6%	5.2%	

Statistically significant differences were observed between individuals not using medication and those using medication in terms of vitamin B12 status in all quarters (p < 0.001) (Table 6). The prevalence of vitamin B12 deficiency was consistently lower among individuals not using medication, ranging from 80 (2.5%) to 90 (3.0%), compared to those using medication, where it ranged from 380 (5.7%) to 435 (6.6%).

**Table 6:** Relationship between vitamin B12 and participant's medication usage in all quarters of the year 2023

Quarters	Vitamin B12 Status		Using medication		Total	P value
			No	Yes		
1^st^ quarter	Normal	N	2527	6145	8672	<0.001
		%	97.2%	93.4%	94.5%	
	Deficiency	N	72	435	507	
		%	2.8%	6.6%	5.5%	
2^nd^ quarter	Normal	N	2877	6023	8900	<0.001
		%	97.4%	93.8%	95.0%	
	Deficiency	N	76	397	473	
		%	2.6%	6.2%	5.0%	
3^rd^ quarter	Normal	N	3167	6286	9453	<0.001
		%	97.5%	94.3%	95.4%	
	Deficiency	N	80	380	460	
		%	2.5%	5.7%	4.6%	
4^th^ quarter	Normal	N	2959	5871	8830	<0.001
		%	97.0%	93.7%	94.8%	
	Deficiency	N	93	393	486	
		%	3.0%	6.3%	5.2%	

## Discussion

The analysis of sociodemographic characteristics and vitamin B12 status across different quarters of 2023 provides valuable insights into the epidemiology of vitamin B12 deficiency within the studied population. Gender distribution revealed a slightly higher representation of females across all quarters, with consistent but statistically insignificant variations between males and females. Age distribution highlighted the prevalence of middle-aged individuals (56-65 years) as the largest demographic group, while those aged <=25 years constituted the smallest proportion. Notably, the majority of patients were Saudi citizens, reflecting the regional demographic composition.

In contrast to the findings reported by Albalawi et al. (2022) [16], whose research identified a notable prevalence of vitamin B12 deficiency among 10.3% of adults, exceeding the threshold rate of 6% proposed by the World Health Organization, our study presents contrasting results regarding vitamin B12 status. Our data analysis revealed that the majority of patients maintained normal vitamin B12 levels throughout the year, with only a small proportion identified as deficient. The prevalence of vitamin B12 deficiency exhibited slight variations across quarters, ranging from 4.6% to 5.5%, indicating no distinct trend of increase or decrease over time.

This observed discrepancy in vitamin B12 deficiency rates could potentially be attributed to the rigorous monitoring protocols implemented in our research setting. Frequent clinic visits and routine vitamin B12 level assessments within the center where our study was conducted may have facilitated early detection and intervention, contributing to the overall maintenance of normal vitamin B12 levels among the majority of patients. These findings highlight the critical importance of continuous monitoring and proactive intervention strategies aimed at addressing and managing vitamin B12 deficiency within the population.

Furthermore, our study underscores the significance of regular assessment and management of vitamin B12 status, particularly among individuals at risk of deficiency. The prevalence rates observed in our study, although lower than those reported by Albalawi et al. (2022) [16], emphasize the need for comprehensive healthcare approaches that prioritize preventive measures and early detection of nutrient deficiencies. By integrating routine vitamin B12 assessments into clinical practice and promoting patient education on dietary and lifestyle factors influencing vitamin B12 status, healthcare providers can effectively mitigate the impact of vitamin B12 deficiency and optimize patient outcomes.

Our study revealed a consistent pattern of higher prevalence of vitamin B12 deficiency among females compared to males across all quarters, with statistical significance observed in the second and fourth quarters. This gender disparity suggests potential biological or lifestyle factors influencing differential vitamin B12 status between genders, warranting further investigation into these underlying mechanisms.

In contrast to the 2018 cross-sectional study by Al Saeed and Baraja [11] in Saudi Arabia, which reported no significant gender difference in vitamin B12 levels, our findings highlight regional or population-specific variations in nutrient status. Understanding these discrepancies is crucial for tailoring healthcare interventions aimed at addressing nutrient deficiencies, particularly among vulnerable populations such as females.

Moving forward, future research should focus on unraveling the specific factors contributing to gender-related disparities in vitamin B12 deficiency prevalence. By gaining deeper insights into these influences, healthcare providers can develop targeted strategies to optimize nutrient status and improve overall health outcomes, especially for women who may benefit from personalized approaches to address nutritional needs effectively.

Age emerged as a significant factor influencing vitamin B12 deficiency in our study, with the <=25 years and 36-45 years age groups consistently exhibiting higher prevalence rates compared to other age groups across all quarters. This finding contrasts with the study by Horvat et al. (2016) [17], which reported a mean age of 64.7 years. The observed age-related variations in vitamin B12 status highlight potential differences in metabolism or dietary intake across different age cohorts, highlighting the importance of tailored interventions to address specific age-related nutritional needs.

Our study's identification of higher prevalence rates of vitamin B12 deficiency among younger individuals (<=25 years) and those in the 36-45 years age group suggests unique considerations for nutritional interventions targeting these populations. Understanding age-related factors influencing nutrient status is crucial for developing effective strategies to optimize vitamin B12 levels and promote overall health across diverse age demographics.

Interestingly, our study found that nationality did not significantly influence vitamin B12 levels, with comparable prevalence rates observed between Saudi and non-Saudi nationals across all quarters. This suggests that vitamin B12 deficiency may not be strongly correlated with nationality within this specific population, highlighting the complex and multifactorial nature of vitamin B12 status. Other factors such as dietary habits, genetic predispositions, healthcare access, and lifestyle choices may play more significant roles in determining individual vitamin B12 levels than nationality alone.

These findings underscore the importance of considering a holistic approach to assessing and addressing nutrient deficiencies, taking into account diverse factors beyond nationality. Future research could explore additional demographic and environmental variables to further elucidate the nuanced influences on vitamin B12 status, ultimately informing targeted interventions and public health strategies aimed at optimizing nutritional health across diverse populations.

The impact of medication usage on vitamin B12 status was particularly notable in our study, with individuals not using medication consistently exhibiting a lower prevalence of deficiency compared to medication users across all quarters. Specifically, the prevalence of vitamin B12 deficiency ranged from 2.5% to 3.0% among individuals not using medication, in contrast to 5.7% to 6.6% among those using medication. This association underscores the potential role of medications in altering vitamin B12 metabolism or absorption, highlighting the importance of closer monitoring of vitamin B12 levels in patients using specific medications known to affect vitamin B12 status.

In contrast, a 2018 cross-sectional study by Al Saeed and Baraja [11] in Saudi Arabia focused on individuals with type 2 diabetes using metformin, with a sample size of 307 metformin-using diabetic patients. Their study reported a vitamin B12 deficiency prevalence of 9.4%, indicating a higher prevalence compared to our findings. This disparity suggests variability in vitamin B12 deficiency rates among individuals using metformin, reflecting the importance of tailored monitoring and management strategies for patients on medications that may impact vitamin B12 levels. Continued research efforts are warranted to further elucidate the relationship between medication use, particularly metformin, and vitamin B12 status to optimize patient care and outcomes in clinical practice.

Study limitations

First, our study was confined to patients from Dr. Sulaiman Al Habib Medical Group (HMG) hospitals, which makes it difficult to generalize the results to Saudi Arabia as a whole. Another limitation of our study is that we couldn’t assess the compliance with metformin treatment during the study, and this may have had an impact on the change in serum vitamin B12 in response to metformin.

In conclusion, the prevalence of vitamin B12 deficiency in diabetic patients on metformin was found to be low in this study. The majority of patients had borderline B12 deficiency, necessitating further investigation and management. The metformin dose was the strongest factor contributing to this deficiency. Further studies are required to assess vitamin B12 levels in relation to metformin to represent the entire Saudi Arabia.

Study strengths

The study provides a thorough and comprehensive analysis of the epidemiology of vitamin B12 deficiency among individuals using metformin. It covers various aspects such as prevalence, risk factors, and implications for patient health. The study includes a substantial number of participants, which enhances the reliability and generalizability of the findings. This large sample size allows for more robust statistical analyses and stronger conclusions. By highlighting the significance of tailored interventions, the study emphasizes personalized healthcare strategies. This approach is essential for optimizing treatment outcomes and addressing individual patient needs effectively.

## Conclusions

In summary, this comprehensive analysis presents pivotal findings regarding the epidemiology of vitamin B12 deficiency among individuals using metformin, underscoring the profound impact of medication on B12 levels. The study's emphasis on tailored interventions that account for gender, age, and specific medication regimens reflects its critical contribution to personalized healthcare strategies. Understanding these factors is crucial as they significantly influence vitamin B12 status, potentially affecting long-term health outcomes in metformin users.

The research not only illuminates the prevalence and risk factors associated with vitamin B12 deficiency but also underscores the necessity for targeted interventions. By highlighting gender and age as influential variables, the study emphasizes the need for healthcare providers to adopt nuanced approaches to managing B12 deficiency in diverse patient populations. Moreover, the recognition of medication usage, particularly metformin, as a significant determinant of B12 status reinforces the importance of proactive monitoring and intervention strategies. Furthermore, this analysis serves as a foundation for future research directions. It calls for more in-depth investigations into the underlying mechanisms through which metformin impacts B12 levels. Unraveling these mechanisms is crucial for developing effective preventive and therapeutic measures aimed at mitigating B12 deficiency risks and optimizing patient care outcomes. Additionally, the study's focus on tailored interventions highlights the potential for personalized medicine approaches to enhance treatment efficacy and patient satisfaction.
